# Saccharin Sulfonamides as Inhibitors of Carbonic Anhydrases I, II, VII, XII, and XIII

**DOI:** 10.1155/2014/638902

**Published:** 2014-09-03

**Authors:** Vaida Morkūnaitė, Lina Baranauskienė, Asta Zubrienė, Visvaldas Kairys, Jekaterina Ivanova, Pēteris Trapencieris, Daumantas Matulis

**Affiliations:** ^1^Department of Biothermodynamics and Drug Design, Institute of Biotechnology, Vilnius University, Graičiūno 8, LT-02241 Vilnius, Lithuania; ^2^Department of Bioinformatics, Institute of Biotechnology, Vilnius University, Graičiūno 8, LT-02241 Vilnius, Lithuania; ^3^Department of Organic Chemistry, Latvian Institute of Organic Synthesis, Aizkraukles 21, Riga LV-1006, Latvia

## Abstract

A series of modified saccharin sulfonamides have been designed as carbonic anhydrase (CA) inhibitors and synthesized. Their binding to CA isoforms I, II, VII, XII, and XIII was measured by the fluorescent thermal shift assay (FTSA) and isothermal titration calorimetry (ITC). Saccharin bound the CAs weakly, exhibiting the affinities of 1–10 mM for four CAs except CA I where binding could not be detected. Several sulfonamide-bearing saccharines exhibited strong affinities of 1–10 nM towards particular CA isoforms. The functional group binding Gibbs free energy additivity maps are presented which may provide insights into the design of compounds with increased affinity towards selected CAs.

## 1. Introduction

Carbonic anhydrases (CAs) belong to the lyase family of enzymes and catalyze the reversible reaction of carbon dioxide hydration to bicarbonate ion and proton. There are 15 CA isoforms in human body: twelve of them are catalytically active [1–3], while three are inactive (CAs VIII, X, and XI). The CA is linked to many diseases such as edema, glaucoma, epilepsy, and cancer. Therefore, CA is an important target for pharmaceutical research [4].

Heterocyclic sulfonamides are the most investigated CA inhibitors. Among them, saccharines play a special role, because they already contain the sulfonamide functionality in the heterocyclic system. Therefore, saccharin itself has shown some binding capacity to several CA isoforms. Saccharin has been previously described as a selective inhibitor of CA IX and CA XII at submicromolar level [5, 6]. The bovine CA II and human erythrocyte CAs I and II have been shown to be inhibited by saccharin [7, 8]. Furthermore, 20 newly prepared N-substituted saccharines have been shown to exhibit higher selective binding to CA IX and CA XII isoforms than saccharin itself [9]. Here, we describe the binding properties of saccharin sulfonamides [10] as CA inhibitors. They exhibited good inhibition properties.

The dissociation constants of synthesized compounds to five CA isoforms (I, II, VII, XII, and XIII) were determined by the fluorescent thermal shift assay (FTSA) and isothermal titration calorimetry (ITC) methods. FTSA (also called ThermoFluor, differential scanning fluorimetry, DSF) [11–17] is a rapid screening method that requires low amounts of protein and is based on the shift of protein melting temperature (*T*_*m*_) that occurs upon ligand binding. The *T*_*m*_ is determined by the change of the fluorescence signal observed upon heat-induced protein unfolding. Isothermal titration calorimetry directly determines the dissociation constant and also the enthalpy and entropy of binding. The enthalpy and entropy are not the subject of this paper. Furthermore, ITC requires larger amounts of protein compared to FTSA and cannot determine very weak or too tight binding. However, these two independent methods complement each other for better accuracy of interaction measurements.

## 2. Results

### 2.1. Binding Results

**Figure 1:**
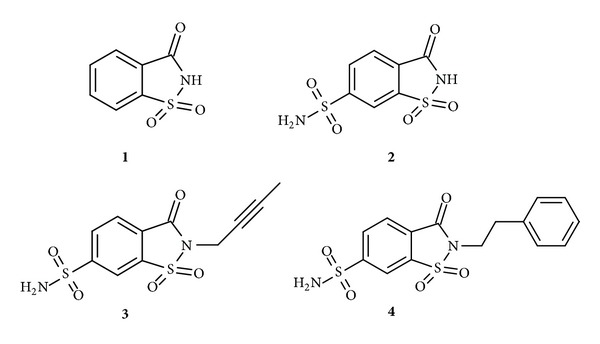
The chemical structures of compounds used in this study. Compound** 1** is saccharin, a secondary sulfonamide. Compound** 2** is sulfonamide-modified saccharin containing both the primary and secondary sulfonamide groups, while compounds** 3** and** 4** contain only the primary sulfonamide groups and are modified on the secondary sulfonamide nitrogen.

**Figure 2:**
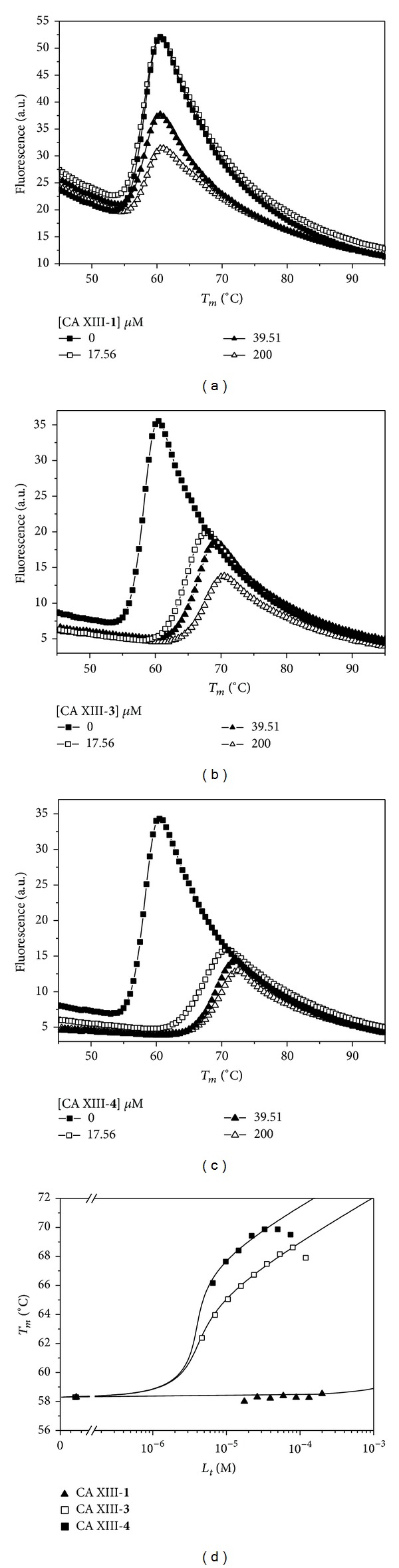
The binding of compounds** 1**,** 3**, and** 4** to CA XIII, determined by the fluorescent thermal shift assay (FTSA). Panels (a)–(c) show the protein melting fluorescence curves as a function of temperature at several added compound concentrations. Saccharin did not exhibit a *T*_*m*_ shift (a) while compounds** 3** (b) and** 4** (c) exhibited a significant shift. Panel (d) shows the resultant three compound dosing curves, the dependencies of the protein melting temperature *T*_*m*_ on the added three compound concentrations. Datapoints are the experimental values obtained from panels (a)–(c) and the solid lines are simulated according to the model as described in Materials and Methods. Experiments were performed at pH 7.0 in sodium phosphate buffer.

**Figure 3:**
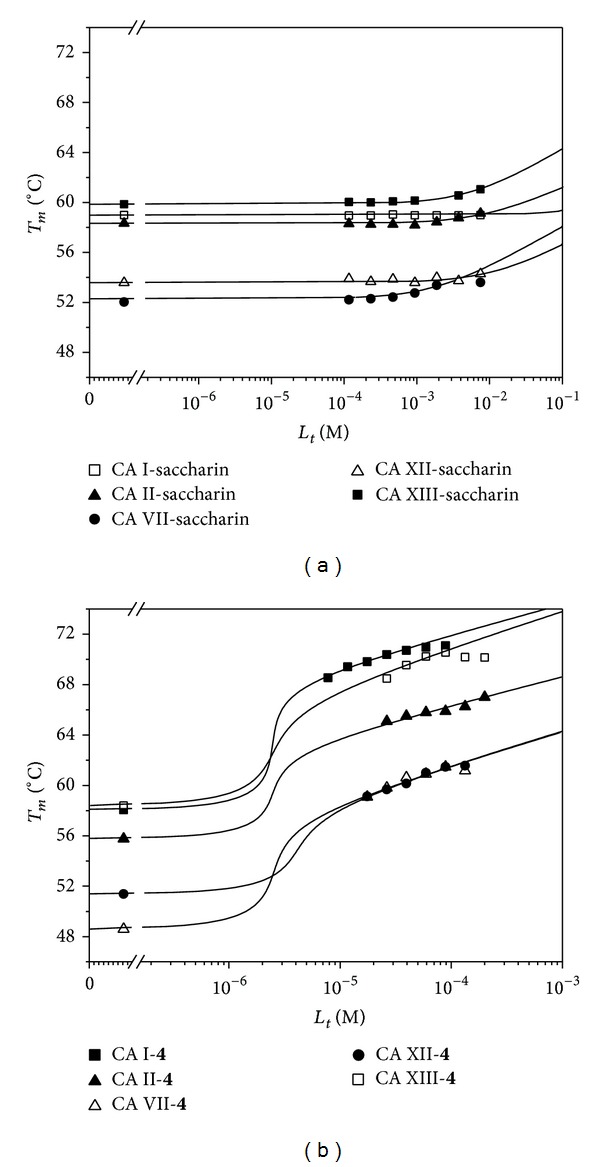
The FTSA dosing curves of compounds** 1** (saccharin, panel (a)) and** 4** (b) binding to CAs I, II, VII, XII, and XIII. Saccharin was dosed up to 7.5 mM and a small *T*_*m*_ shift was observed for all CAs except CA I. Compound** 4** was dosed up to 200 *μ*M with a significant shift. The determined dissociation constants for all compounds are listed in Table 1. There was no added DMSO while dosing saccharin and there was 2% (v/v) final DMSO concentration while dosing** 4**. This explains the reduced *T*_*m*_ of the protein in the absence of compound with DMSO (b) compared to (a) that in the absence of DMSO.

The binding of four saccharin sulfonamides (including saccharin itself, chemical structures shown in Figure 1) to five isoforms of human recombinant catalytic domains of carbonic anhydrases (CAs) was determined by the fluorescent thermal shift assay (FTSA) and isothermal titration calorimetry (ITC).  Figure 2 shows an example of the FTSA data compounds** 1**,** 3**, and** 4** binding to CA XIII. Figures 2(a), 2(b), and 2(c) show the thermal denaturation curves of CA XIII in the presence of various saccharin** 1** and saccharin sulfonamides** 3 **and** 4** concentrations. There was no shift of the melting temperature when saccharin was added to 200 *μ*M concentration (Figures 2(a) and 2(d)). The shift became visible only at higher saccharin concentrations (see Figure 3(a)). However, the shift was significant for the saccharin sulfonamides** 3 **and** 4** (Figures 2(b) and 2(c)).  Figure 2(d) shows the dosing curves and the melting temperatures as a function of added compound concentrations. Lines were simulated as described in Materials and Methods.

Figure 3 shows the dosing curves of the least potent compound** 1** (saccharin) and the most potent compound** 4** binding to all five tested CA isoforms. There is weak shift exhibited by saccharin (**1**) only at highest concentrations around 1–10 mM, while a significant shift of the melting temperature with compound** 4** was observed. However, visual comparison of the affinities is complicated because the melting temperatures of all five CA isoforms are different, varying from about 49°C (CA VII) through 58°C (CAs I and XIII).

The observed dissociation constants *K*_*d*_s for all four compounds binding to the five CA isoforms as determined by the FTSA are listed in Table 1. Saccharin sulfonamide derivatives bound CAs with nanomolar affinities. The affinity of compound** 4** reached 330 pM for CA I and 25 nM for CA XII (Table 1). Compound** 2**, however, exhibited weakest binding of the three saccharin sulfonamides. Its affinity for CA I was only about 3.0 *μ*M but reached about 200 nM for CA XIII.

Saccharin has been previously demonstrated to inhibit numerous CAs, especially CA VII. Our results confirm that CA VII bound saccharin most strongly of the five tested isoforms. However, our determined dissociation constants are in the range of about 1–10 mM and thus are significantly weaker than some previously determined affinities. The FTSA determined that CA VII bound saccharin with the *K*_*d*_ of 1.0 mM; CA XIII, 2.0 mM; CA II, 2.9 mM; CA XII, 5.9 mM; and CA I did not exhibit any detectable shift up to 7.5 mM added saccharin; thus, its *K*_*d*_ is weaker than 10 mM.

**Figure 4:**
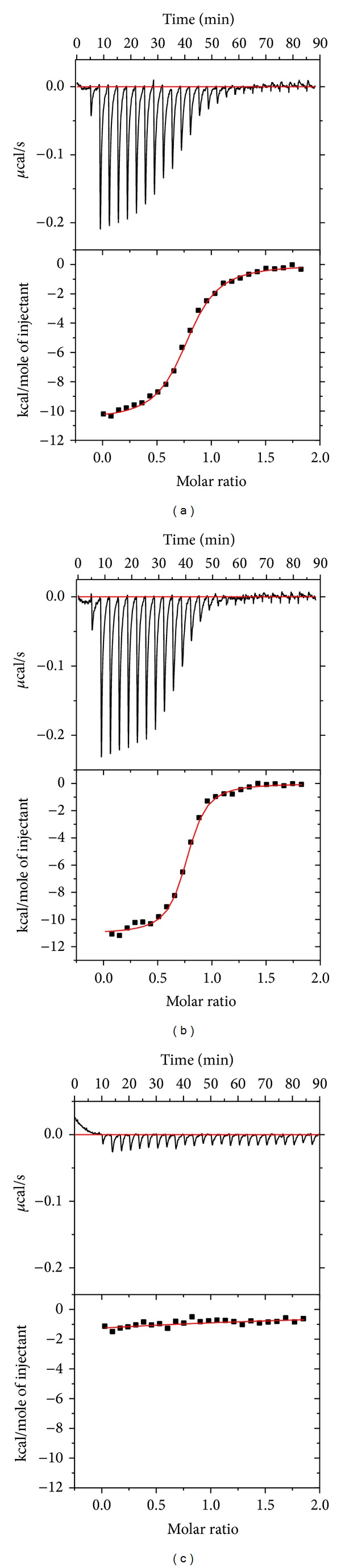
Isothermal titration calorimetry (ITC, 37°C) binding curves for compound** 2** to CA II (panel (a), *K*_*d*_ = 290 nM) and CA XIII (panel (b), *K*_*d*_ = 120 nM) and saccharin (**1**) to CA II (panel (c), *K*_*d*_ > 10^−4 ^M, could not be accurately determined). Table 2 lists the dissociation constants obtained by ITC for the tested compounds binding to all five CAs.

In order to confirm the FTSA measurements, all four compounds binding to five CA isoforms were also measured by the isothermal titration calorimetry (ITC).  Figure 4 shows representative ITC data of compound** 2** binding to CA II and CA XIII and saccharin binding to CA II. The ITC data for all compounds and CAs are listed in Table 2. Dissociation constants obtained by ITC were essentially confirming the FTSA results. However, the most potent compounds that exhibited affinity stronger than 20 nM by FTSA bound too tight to CA for accurate *K*_*d*_ determination by ITC. As described in [13], the Wiseman *c* factor must be between 10 and 100 for precise *K*_*d*_ determination by ITC (could be still determined in the range of 5–1000 with lower accuracy). In titrations of compound** 4** with CAs I, II, and VII, the *c* value surpassed 1000. Therefore, such determinations could not be confirmed by ITC. Saccharin itself did not exhibit any binding by ITC as seen in Figure 4(c).

**Figure 5:**
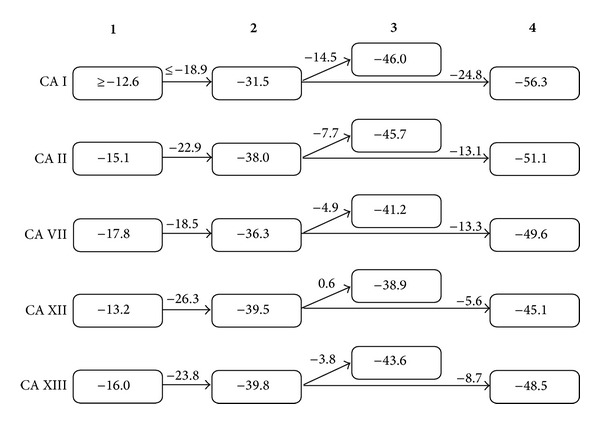
The thermodynamics of saccharin (**1**) and saccharin derivatives (**2**–**4**) binding to five CA isoforms, determined by FTSA at pH 7.0 and 37°C. The numbers within the shapes represent the Gibbs free energies of binding to a particular CA while the numbers on arrows show the differences in Δ*G* between two compounds that are most similar in chemical structure (kJ/mol). These differences represent the functional group contributions to the overall binding thermodynamics.

Dissociation constants were recalculated to the Gibbs free energies of binding in order to make easy comparison between different compound structures in the order of increasing affinities.  Figure 5 shows such Gibbs free energy additivity scheme for all five CA isoforms. The addition of the alkyne group (compound** 3** versus** 2**) significantly strengthened the binding to CAs I, II, VII, and XIII but not towards CA XII. However, consecutive replacement of the alkyne group with the phenyl group (compound** 4**) increased the binding affinity towards all five CAs. The energetic contribution was greatest for CA I (−14.5 and −24.8 kJ/mol, resp.). Compound** 4** exhibited the largest affinities towards all five CA isoforms. However, affinity itself does not automatically increase selectivity towards a particular CA isoform. Selectivity towards a particular CA isoform is often a goal for a drug that would not have side effects by inhibiting a nondesired isoform. These Gibbs free energy diagrams helped design a very potent compound** 4** that is also quite selective towards CA I isoform.

### 2.2. Docking Results

To explore the ligand-protein interactions for the complexes which did not have available X-ray structures, compounds** 3** and** 4** were docked in the active site of CA II using Vdock docking program [18, 19]. Here, we assumed that sulfonamide group is bound to the active site zinc as is indicated from the binding results. Furthermore, similar to our previous study [20], we used constrained docking to achieve reasonable results. A spatial constraint was imposed on the zinc-bound nitrogen to mimic a strong coordination between the zinc and sulfonamide nitrogen. An additional constraint was imposed on the sulfur-benzene ring bond torsional angle to maintain the correct rotamer. The main reason for the torsional constraints was an apparent lack of correct torsional potentials for the bond connecting the bulk of ligand with the sulfonamide group [21, 22].

The preliminary docking showed that the benzene ring had a strong tendency towards aligning with the sulfonamide S–N bond, while a visual inspection of the structures available in the PDB showed that many structures are staggered or nearly aligned with one of the S=O bonds. To verify this, we performed a survey of the available PDB [23] structures. A search revealed 115 CA structures with 124 ligand conformations in which the active center zinc-bound sulfanilamide group is attached to a benzene ring with both hydrogen substituents at the orthoposition. We explored the statistics of the torsional angles that the benzene ring plane makes with respect to the sulfonamide oxygens. The vast majority of the angles were in a narrow range characterized by an approximately staggered conformation on one end and an eclipsed rotamer in which the benzene ring was aligned with one of the S=O bonds on the other end of the range. Out of the 124 ligand conformations, there were only five outliers (PDB ID: 3ca2, 3b4f, 3nj9, 3p3h, and 3p3j) in which the benzene ring was aligned with the sulfonamide nitrogen. These five ligands are characterized by bulky substituents at the meta- or paraposition of the benzene ring. Because of the small number of the outliers and because of a small size of compounds** 3** and** 4, **we felt these outliers can be rather safely not taken into statistics for our purposes. Among the rest of the 119 ligand conformations, the average torsion angle between the S=O and the plane of the benzene ring was 14.7° (σ = 7.8°).

**Figure 6:**
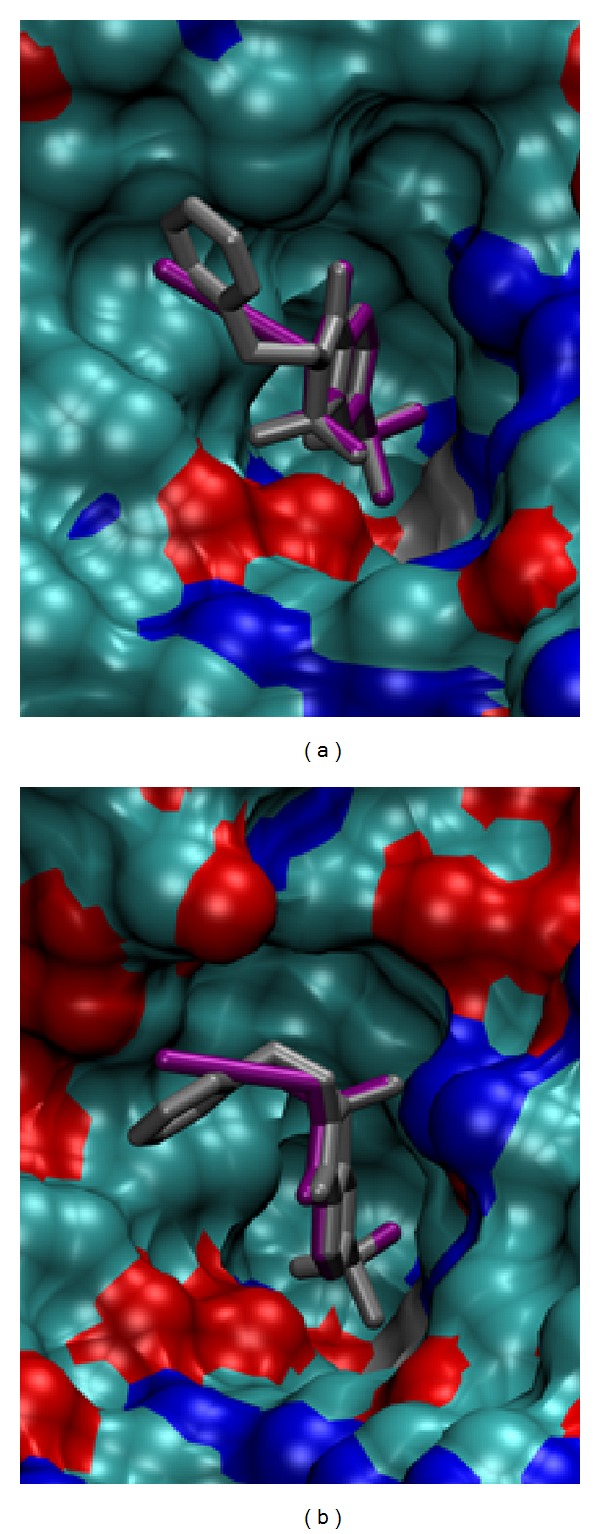
The dockingof compounds** 3** (purple) and** 4** (gray) into the active site of CA II (a) and CA XII (b). The solvent accessible surface of the protein is displayed to demonstrate how well the ligand fits the active site of CA II. Compound tails are arranged quite differently in CAs II and XII. The protein surface is colored to show oxygen atoms (red), nitrogen atoms (blue), and zinc atom (gray), and the rest is in cyan-blue.

Based on the findings, we chose as a reference a compound having a similar ring structure to** 3**-**4,** indane-5-sulfonamide ligand (PDB ID: 2qoa) [24] with O=S–C–C torsion angle 18.0°, which is close to the average described above. A ±15° constraint around the indane-5-sulfonamide value was imposed on the corresponding dihedral angles of** 3**-**4 **during the docking. The docking results are shown in Figure 6. The hydrophobic tails of the ligands** 3-4 **lie in the hydrophobic groove framed by residues Phe131, Val135, Pro202, and Leu204. The side chain of Thr200 forms the hydrogen bonds with the sulfonyl oxygens in the thiazole ring of** 3** and** 4**.

Compounds** 3**-**4 **were also docked into CA XII (PDB ID: 1jd0 [25]) to possibly rationalize a relatively poor binding of** 3** and** 4** compared to CA II. Somewhat unexpectedly, these two compounds docked in a reverse binding mode compared to CA II: in CA XII the benzothiazole ring of these ligands is flipped (Figure 6(b)). In the reverse binding mode, one of the oxygen atoms of the sulfonyl group in the thiazole ring forms a hydrogen bond with Gln92 side chain. However, this hydrogen bond is likely to be much weaker compared to the complex with CA II because the hydrogen bond length is larger by more than 0.4 Å in the complex with CA XII as compared to CA II.

## 3. Discussion

Sulfonamides are the most investigated inhibitors of carbonic anhydrases. Primary SO_2_NH_2_ group binds to zinc atom in the active site and inhibits the protein catalytic activity. However, it is desirable to make compounds that would be good inhibitors of selected CA isoforms and would not bear the sulfonamide group. One such compound is saccharin that was previously shown to be a potent and quite selective inhibitor towards selected CA isoforms [5]. Furthermore, the crystal structure of saccharin bound to CA II has been determined.

This study confirmed that saccharin bound four of the tested five CA isoforms by direct biophysical techniques such as the fluorescent thermal shift assay. Interestingly, the binding of saccharin modified with sulfonamide group exhibited strong binding to all tested CA isoforms. Saccharin binding was carefully measured several times and could be detected for any of the tested CA isoforms only at concentrations of 1–10 mM. The *K*_*d*_s for saccharin binding to four CA isoforms ranged from 1.0 to 5.9 mM (except CA I where there was no shift detected). This result significantly contradicts earlier finding by Köhler et al. and D'Ascenzio et al., where all tested 14 isoforms of CA bound saccharin with nanomolar to micromolar affinity [5, 9]. The CA VII has been shown to bind saccharin with 10 nM affinity [5]. Our results indicate that the affinity is 100,000-fold weaker. However, our results confirm that saccharin bound CA VII, the strongest of the five tested isoforms. Therefore, selectivity towards CA VII is shown by both FTSA and inhibition methods. Isothermal titration calorimetry confirmed that saccharin bound all tested CAs with the *K*_*d*_ weaker than 100 *μ*M.

There is no clear explanation for such significant discrepancy between the binding measurements by FTSA-ITC and inhibition of activity measurements by stopped-flow CO_2_ hydration assay. However, different approaches sometimes yield different results and it may be necessary to further investigate this reaction by other techniques.

The thermodynamics-structure correlation diagrams, as shown in Figure 5, could help design compounds with desired properties such as increased affinities or increased selectivity toward a desired CA isoform. However, it should be kept in mind that the diagrams in Figure 5 and the values in Tables 1 and 2 represent only the observed *K*_*d*_s that are valid exclusively for pH 7.0 and 37°C. These values depend on pH because there are linked protonation reactions observed upon compound binding to CA [26, 27]. In order to better understand the structure-thermodynamics correlations, it would be important to obtain the intrinsic values of binding by subtracting the contributing protonation reactions. However, such subtraction is not the subject of this paper because the actual affinities occurring at pH 7.0 are the observed values and it is important to compare them with the ones obtained by other methods such as stopped-flow CO_2_ hydration assay [5, 9].

Saccharine contains only a secondary sulfonamide group that apparently binds CAs significantly weaker than modified compounds bearing primary sulfonamide groups. Their binding is expected to be quite different and thus exhibits significantly different affinities for CAs. Compound** 2** bears both the primary and secondary sulfonamide groups, but it is expected to bind through its primary sulfonamide to the zinc of CA as confirmed by docking. Furthermore, it bound significantly stronger than saccharin itself.

Compounds** 3** and** 4** are the strongest binders of the four tested compounds towards all CAs. It was interesting to see if some of their structural moieties could be used for the design of compounds with even greater affinity or increased selectivity towards a desired CA isoform. Docking showed that the compounds bound in a quite different structural arrangement when comparing CA II and CA XII docked structures. This indicates that these and similar functional groups could be used to further increase the selectivity towards, for example, cancer related CA XII.

The affinity of several millimolar is sufficient to detect binding by X-ray crystallography if the added compound concentration is of the order of 1 mM. Therefore, there is no contradiction between our results that show weak saccharin binding and crystallographic structures that demonstrate saccharin bound in the active site of a CA.

## 4. Materials and Methods

### 4.1. Organic Synthesis of the Compounds

Saccharin (**1**) was purchased while compounds** 2**–**4** were synthesized as previously described in [10]. Figure 1 shows the chemical structures of saccharin (**1**) and the three saccharin sulfonamide derivatives (**2**,** 3**, and** 4**) that are the subject of this paper.

### 4.2. Protein Preparation

Carbonic anhydrase isoforms I, II, VII, XII, and XIII were expressed and purified as previously described: CA I in [28], CA II in [29], CAs VII and XIII in [30], and CA XII in [26].

### 4.3. Determination of Compound Binding to CA Isoforms

#### 4.3.1. Fluorescent Thermal Shift Assay

The fluorescent thermal shift assay (FTSA) measurements were performed in a Corbett Rotor-Gene 6000 (QIAGEN Rotor-Gene Q) instrument using the blue channel (excitation 365 ± 20, detection 460 ± 15 nm). The samples (20 *μ*L volume) contained 5–10 *μ*M protein, 0–200 *μ*M compound, and 50 *μ*M ANS (8-anilino-1-naphthalene sulfonate) in 50 mM sodium phosphate buffer (pH 7.0), 50 mM NaCl, and 0 or 2% DMSO (concentration in the final assay, v/v). The applied heating rate was 1°C/min. Data analysis was performed as previously described [28].

#### 4.3.2. Isothermal Titration Calorimetry

Isothermal titration calorimetry (ITC) experiments were performed using VP-ITC instrument (Microcal, Inc., Northampton, USA) with 4–10 *μ*M protein solution in the cell and 40–100 *μ*M of the ligand solution in the syringe. A typical experiment consisted of 25 injections (10 *μ*L each) within 3 min intervals. Experiments were performed at 37°C in 50 mM sodium phosphate buffer containing 50 mM NaCl at pH 7.0, with a final DMSO concentration of 2%, equal in the syringe and the cell.

#### 4.3.3. Docking

Docking was performed using Vdock program [18, 19]. Structures 1jd0 [25] and 4g0c [31] were used to build CA II and CA XII receptors, respectively. The ligands were built using Avogadro v. 1.1.0 [32]. Force fields CHARMM22 [33] and CHARMm with Momany-Rone charges [34] were used to model the protein and ligand, respectively. The ligand parameters were generated using Discovery Studio Visualizer v. 3.5 (Accelrys Software Inc., San Diego, CA). Sulfonamide nitrogen was fixed at an X-ray position by setting it as a translational center and placing it into the translational box whose dimension was reduced to zero. The solvent effect during docking was modeled with the distance-dependent dielectric approximation, *ε*_*ij*_ = 4*r*_*ij*_ [35]. One hundred minima were generated (instead of the default 20) and the genetic algorithm was switched off during docking to ensure a more thorough search of the conformational space. The docked structures were graphically represented using VMD [36].

## 5. Conclusions

The saccharin sulfonamides exhibited nanomolar and subnanomolar affinities toward selected CA isoforms. However, unmodified saccharin bound the tested CAs with only 1–10 mM affinity. Saccharin is a secondary sulfonamide and bound CAs significantly weaker than saccharin sulfonamide derivatives that are primary sulfonamides. The Gibbs free energies of binding show the functional group influence on the binding constants of saccharin and its sulfonamide derivatives.

## Figures and Tables

**Table 1:** Compound dissociation constants of human recombinant carbonic anhydrase isoforms I, II, VII, XII, and XIII, as determined by the fluorescent thermal shift assay (FTSA, 37°C, pH 7.0). Acetazolamide (**AZM**) was used as a control. Saccharin bound with the *K*_*d*_ of 1 to over 10 mM while compound **4** reached the affinity of 0.3 to 25 nM.

Compound	Dissociation constants *K*_*d*_ (nM) and CA isoforms				
	CA I	CA II	CA VII	CA XII	CA XIII
**1 (saccharin)**	>10,000,000	2,900,000	1,000,000	5,900,000	2,000,000
**2**	3000	400	770	220	200
**3**	18	20	110	290	45
**4**	0.33	2.5	4.3	25	6.7
**AZM**	1400	38	17	130	50

Uncertainties of the FTSA measurements are approximately 1.6-fold in *K*_*d*_.

**Table 2:** Compound dissociation constants of human recombinant CA isoforms I, II, VII, XII, and XIII, as determined by ITC (37°C, pH 7.0). Acetazolamide (**AZM**) was used as a control. The affinity range of ITC determinations is quite narrow. At our experimental conditions, 5–10 *µ*M CA in the calorimeter cell, the range may cover 1000–50 nM affinities (for the Wiseman factor of 100 to 10). With some approximation, the range could be expanded to cover affinities of 100 *µ*M to 20 nM. These ITC measurements essentially confirm the FTSA results.

Compound	Dissociation constants *K*_*d*_ (nM) and CA isoforms				
	CA I	CA II	CA VII	CA XII	CA XIII
**1**	>100,000	>100,000	>100,000	>100,000	>100,000
**2**	1400	290	560	99	120
**3**	28	56	160	250	47
**4**	<20	<20	<20	40	<20
**AZM**	810	46	63	130	60

Uncertainties of the ITC measurements are approximately 2.0-fold in *K*_*d*_.
